# Acute kidney injury and beyond

**DOI:** 10.12861/jrip.2012.01

**Published:** 2012-01-01

**Authors:** Hamid Nasri

**Affiliations:** ^1^Department of Nephrology, Division of Nephropathology, Isfahan University of Medical Sciences, Isfahan, Iran.

**Keywords:** Acute kidney injury, Chronic renal failure, Mortality

**Implication for health policy/practice/research/medical:**

Recent epidemiological investigations had demonstrated the increased mortality link with acute kidney injury and importantly suggest a connection to the subsequent development of chronic renal failure.

Acute kidney injury (AKI) is a complex disorder that happens in a variety of conditions with clinical manifestations extending from a minimal elevation in serum creatinine to anuric kidney insufficiency (1-3). It is usually under-recognized and is accompanied with severe outcomes. AKI is associated with length of hospital stay, increased mortality, and costs. Recent evidences indicates that even minor changes in serum creatinine are linked with increased in-patient mortality (2-5). Over the last decades, AKI has been the center of extensive clinical and basic research works. In spite of the noteworthy progress created in understanding the biology and mechanisms of AKI in animal models, interpretation of this findings into improved management and consequences for patients has been inadequate (2-6). It is of particular importance that, recent epidemiological investigations had demonstrated the wide variation in etiologies and risk factors, explain the increased mortality link with AKI and importantly suggest a connection to the subsequent development of chronic renal failure and subsequent dialysis dependency (3-6). Therefore, AKI has been incriminated as an independent risk factor for the development of chronic renal failure. Thus AKI is not uncommon and it is frequently under-recognized and is associated with severe outcomes. This adverse outcome might be due to the late recognition of AKI when the elevation of serum creatinine level is used. Several proteins and biochemical markers developed as sensitive and specific biomarkers capable of identifying kidney damage early and proved to be hopeful biomarkers as signs of AKI in recent investigations (4-8). A panel of urinary biomarkers was found and may improve the early detection of postoperative AKI. These include neutrophil gelatinase associated lipocalin (NGAL), cystatin C, N-acetyl-β-D-glucosaminidase (NAG), IL-18 and kidney injury molecule-1 (KIM-1). However, larger prospective studies are essential to validate the temporal expression pattern of various urinary biomarkers for early recognition of AKI, how to combine multiple biomarkers for early finding of AKI, and how this temporal course connects to the onset, severity, and outcome of AKI (4-10). Thus still larger experimental and clinical investigations needs to find significance of these biomarkers.

## Author’s contribution

HN is the single author of the manuscript.

## Conflict of interests

The author declared no competing interests.

## Ethical considerations

Ethical issues (including plagiarism, misconduct, data fabrication, falsification, double publication or submission, redundancy) have been completely observed by the authors.

## Funding/Support

None.
